# Prevalence of chronic pain in LTCs and multimorbidity: A cross-sectional study using UK Biobank

**DOI:** 10.1177/26335565211005870

**Published:** 2021-12-21

**Authors:** Ross McQueenie, Bhautesh Dinesh Jani, Stefan Siebert, Philip McLoone, Colin McCowan, Sara Macdonald, Frances S Mair, Barbara I Nicholl

**Affiliations:** 1 Institute of Health and Wellbeing, MVLS, University of Glasgow, Glasgow, UK; 2 Institute of Infection, Immunity and Inflammation, MVLS, University of Glasgow, Glasgow, UK; 3 School of Medicine, University of St Andrews, Andrews, UK

**Keywords:** Chronic pain, multimorbidity, long-term conditions, prevalence

## Abstract

**Objectives:**

Chronic pain is often experienced alongside other long-term conditions (LTCs), yet our understanding of this, particularly in relation to multimorbidity (≥2 LTCs) is poor. We aimed to examine associations between the presence/extent of chronic pain with type/number of LTCs experienced.

**Methods:**

We examined the relationship between number/type of LTCs (N = 45) in UK Biobank participants (n = 500,295) who self-reported chronic pain lasting ≥3 months in seven body sites or widespread. Relative risk ratios (RRR) for presence/extent of chronic pain sites were compared using logistic regression adjusted for sociodemographic (sex/age/socioeconomic status) and lifestyle factors (smoking/alcohol intake/BMI/physical activity).

**Results:**

218,648 participants self-reported chronic pain. Of these, 69.1% reported ≥1 LTC and 36.2% reported ≥2 LTCs. In 31/45 LTCs examined, >50% of participants experienced chronic pain. Chronic pain was common with migraine/headache and irritable bowel syndrome where pain is a primary symptom, but also with mental health conditions and diseases of the digestive system. Participants with >4 LTCs were over three times as likely to have chronic pain (RRR 3.56, 95% confidence intervals (CIs) 3.44–3.68) and 20 times as likely to have widespread chronic pain (RRR 20.13, 95% CI 18.26–22.19) as those with no LTCs.

**Conclusions:**

Chronic pain is extremely common across a wide range of LTCs. People with multimorbidity were at higher risk of having a greater extent of chronic pain. These results show that chronic pain is a key factor for consideration in the management of patients with LTCs or multimorbidity.

## Introduction

Chronic pain, defined as pain lasting for 3 months or more, is a common and limiting condition worldwide.^1–3^ Chronic pain may be experienced in relation to a specific body site, e.g. low back pain, or be present in multiple sites of the body, including widespread pain, defined as pain in at least four of five body sites across three quadrants of the body and the spine.^4^ A recent systematic review and meta-analysis reported that approximately 43% of adults in the UK live with chronic pain, and between 11% and 17% report disabling widespread pain.^5^ Chronic pain is frequently experienced alongside other long-term conditions (LTCs).^6^

Multimorbidity is defined as the presence of two or more LTCs.^7^ Treatment plans for patients with multimorbidity are often complex and can be challenging, often resulting in an increased treatment burden due to the workload of self-management,^8^ increased hospital admissions^9^ and increased potential for polypharmacy,^10^ and lower quality of life.^11^ Despite this, current treatment and clinical guidelines largely focus on the management of individual LTCs.

While there is some literature to show that chronic pain and LTCs often co-exist,^12,13^ there is much less information about which LTCs are most often associated with chronic pain and of the relationship of multimorbidity with chronic pain. Some recent studies have investigated the effect of chronic pain in the presence of specific individual LTCs, for example in major depression and bipolar disorder,^14^ cardiovascular disease,^15^ and rheumatoid arthritis.^16^ However, studies examining prevalence of comorbidities in those with chronic pain have generally involved small sample sizes^17^ or have only reported on the prevalence of chronic pain as a comorbidity across a limited range of conditions^17^ and we know insufficient information about chronic pain in the context of multimorbidity or as a comorbidity to a wider range of LTCs. This matters because chronic pain is often poorly managed and can result in major functional limitations and reduced quality of life (QoL), which may exacerbate or be exacerbated by the presence of other LTCs.^18^

The aim of this study was to examine the association between both the presence and extent (number of sites) of chronic pain and the type and number of LTCs experienced in a large cohort of middle-older aged adults. We hypothesised that those with a greater number of LTCs would be more likely to have chronic pain and to have more extensive chronic pain.

## Methods

### Study design and participants

We examined baseline self-reported health from UK Biobank assessment centre visits recorded between 2006 and 2010. This dataset contains information on 502,503 participants who attended recruitment centres in Scotland, England and Wales to complete self-administered touch screen and nurse-guided questionnaires. The data collected covered a range of sociodemographic, lifestyle and self-reported LTC questions. This study was covered by the generic ethics approval for UK Biobank studies from the NHS National Research Ethics Service (16/NW/0274) and conducted as part of UK Biobank project 14151.

### Classification of chronic pain and LTCs

Participants were asked about pain in the touchscreen questionnaire; they were first asked if they had any pain in the last month that interfered with their usual activities in the following body sites: head, face, neck/shoulder, back, abdomen, hip, knee or ‘pain all over the body’ (referred to in this study as widespread pain). Participants who answered affirmatively to pain in any of these sites were then asked whether this pain had lasted for 3 months or longer. Participants answering yes to any of these sites were considered to have chronic pain. Using this information, a categorical variable of number of chronic pain sites was created with the following categories: 0, 1, 2–3, ≥4 or ‘widespread pain’; used to ascertain the extent of pain reported.

Self-report LTC information was generated from responses to a nurse-led questionnaire. Participants answered whether they had been told by a doctor that they had a serious illness or disability, and if so, what they were. Of these, 45 individual self-reported LTCs were chosen for inclusion in this study based on an adaptation of N = 43 conditions used in previous UK Biobank studies of multimorbidity.^14,19^ Supplementary Table 1 lists all LTCs examined.

LTCs were examined both as prevalence of individual LTCs experienced alongside chronic pain, and as the extent of multimorbidity, by counting LTCs and categorising into: 0, 1, 2–3 or ≥4 LTCs.

### Covariates

Age was categorised into groups: 37–49, 50–59, 60–72 years old. Socioeconomic status was measured using Townsend score, a measure of UK deprivation,^20^ and categorised into quintiles ranging from 1 (least deprived) to 5 (most deprived). Smoking status was categorised into never-smokers and current/previous smokers. Alcohol intake was categorised by frequency of intake (Never or special occasions only, 1–3 times per month, or at least once per week). BMI was categorised into groups (<18.5, 18.5–24.9, 25–29.9, 30–34.9, 35–39.9 and ≥40 kg/m^2^) based on measurements taken during the baseline assessment then categorised based on WHO obesity guidelines.^21^ Physical activity was categorised into ‘none’, ‘low’, ‘medium’ and ‘high’ based on the responses to the UK Biobank physical activity questionnaire.

### Statistical analysis

Descriptive analysis was used to examine demographic and lifestyle factors associated with the presence (yes to one of more sites of chronic pain) and number of chronic pain sites (extent of chronic pain). We used cross-tabulations of number of chronic pain sites by age, gender, socioeconomic deprivation (Townsend score), smoking status, frequency of alcohol consumption, BMI, and physical activity. We used a χ2 test to determine whether there was a significant difference between pain groups. To measure the relative risk of the presence and extent of chronic pain in relation to multimorbidity count, we used a multinomial logistic regression model controlling for demographic and lifestyle variables as described above. Results were considered significant if p < 0.01. Participants who did not answer questions on chronic pain were excluded from analysis (N = 2190; 0.44%). All analysis was conducted using R version 3.2.3.

## Results

A total of N = 500,295 participants provided complete information on chronic pain and were included in this analysis. Participants were aged between 37 and 73 (mean age was 56.53 (standard deviation (SD) = 8.09)); 45.6% (N = 228,069) of participants were male.

### Demographic and lifestyle factors

Table 1 shows demographic and lifestyle factors in relation to the presence and extent of chronic pain reported. 218,648 (43.7%) participants stated that they experienced chronic pain in at least one site (head, hip, knee, shoulder, head, abdomen or widespread) for 3 or more months. 115,193 (23.0%) reported one site of chronic pain, 81,406 (16.3%) 2–3 sites, 14,924 (3.0%) 4–7 sites, and 7125 (1.4%) reported widespread pain. Participants with a greater number of chronic pain sites were more likely to be female, have lower socioeconomic status, were current or previous smokers, never drank alcohol or on special occasions only, be obese, and participate in moderate amounts of physical activity.

**Table 1.** Lifestyle factors, demographic factors, and extent of multimorbidity in participants with and without chronic pain, by presence and extent of chronic pain.

	Presence of chronic pain		Extent of chronic pain			
	No (%) (N = 281,647; 56.3%)	Yes* (%) (N = 218,648; 43.7%)	One site (%) (N = 115,193; 23.0%)	Two to three sites (%) (N = 81,406; 16.3%)	Four to seven sites (%) (N = 14,924; 3.0%)	Widespread pain (%) (N = 7125; 1.4%)
Age						
37–49	68776 58.6%	48487 41.4%	26633 22.7%	17431 14.9%	3083 2.7%	1340 1.1%
50–59	93134 55.9%	73343 44.1%	38037 22.8%	27111 16.3%	5489 3.3%	2706 1.6%
60–73	119737 55.3%	96818 44.7%	50523 23.3%	36864 17.0%	6352 2.9%	3079 1.4%
Sex						
Female	146497 53.8%	125729 46.2%	62649 23.0%	48686 17.9%	9852 3.6%	4542 1.7%
Male	135150 59.3%	92919 40.8%	52544 23.0%	32720 14.3%	5072 2.2%	2583 1.1%
Townsend score (level of deprivation)						
1 (least deprived)	60049 59.8%	40375 40.2%	22833 22.7%	14607 14.5%	2069 2.1%	866 0.9%
2	58333 58.4%	41524 41.6%	23126 23.2%	15140 5.2%	2334 2.3%	924 0.9%
3	57147 57.1%	42937 42.9%	23390 23.4%	15804 15.8%	2588 2.6%	1155 1.2%
4	55934 56.0%	43983 44.0%	22869 22.9%	16464 16.5%	3161 3.2%	1489 1.5%
5 (most deprived)	49858 50.2%	49536 49.8%	22837 23.0%	19276 19.4%	4740 4.8%	2683 2.7%
Smoking status						
Current or Previous	119914 53.2%	105628 46.8%	53543 23.7%	40454 17.9%	7913 3.5%	3718 1.6%
Never	160798 58.9%	112092 41.1%	61229 22.4%	40574 14.9%	6931 2.5%	3358 1.2%
Frequency of alcohol intake						
Never or special occasions only	46924 47.8%	51231 2.2%	22231 22.6%	20346 20.8%	5482 5.6%	3172 3.2%
One to three times a month	29975 53.8%	25771 46.2%	12901 23.1%	10045 18.0%	1978 3.5%	847 1.5%
One to four times a week	143735 58.8%	100582 41.2%	56518 23.1%	36236 14.8%	5502 2.2%	2326 0.9%
Daily or almost daily	60815 59.8%	40841 40.2%	3443 23.1%	14686 18.0%	1945 .5%	767 0.8%
BMI						
underweight < 18.5	1611 61.6%	1003 38.4%	521 19.9%	355 13.6%	83 3.2%	44 1.7%
normal weight 18.5–24.9	97908 62.4%	59035 37.6%	33996 21.7%	20730 13.2%	2996 1.9%	1313 0.8%
overweight 25–29.9	122651 57.4%	90852 42.5%	49141 23.0%	33629 15.7%	5514 2.6%	2568 1.2%
obese ≥30	58264 46.8%	66329 53.2%	30919 24.8%	26174 21.0%	53057 2.4%	3057 2.4%
Physical activity						
None	13917 42.6%	18740 57.4%	7257 2.2%	7413 22.7%	2427 7.4%	1643 5.0%
Low	8373 44.3%	10518 55.7%	4587 24.3%	4228 22.4%	1083 5.7%	620 3.3%
Medium	223563 56.9%	169112 43.1%	91214 23.2%	63119 16.1%	10454 2.7%	4325 1.1%
High	33137 66.2%	16889 33.8%	10805 21.6%	5364 10.7%	572 1.1%	148 0.3%
Number of long-term conditions						
0	125067 65.0%	67258 35.0%	41809 21.7%	22256 11.6%	2329 1.2%	864 0.4%
1	93594 56.6%	71717 43.3%	39599 23.9%	26431 16.0%	3873 2.3%	1814 1.1%
2–3	56989 46.6%	65337 53.4%	29490 24.1%	26744 21.9%	6136 5.0%	2967 2.4%
≥4	5346 28.5%	13427 71.5%	3945 21.0%	5613 29.9%	2466 13.1%	1403 7.5%

*Participants with at least one site of chronic pain.

### Individual LTCs, extent of multimorbidity and chronic pain

**Figure 1.**
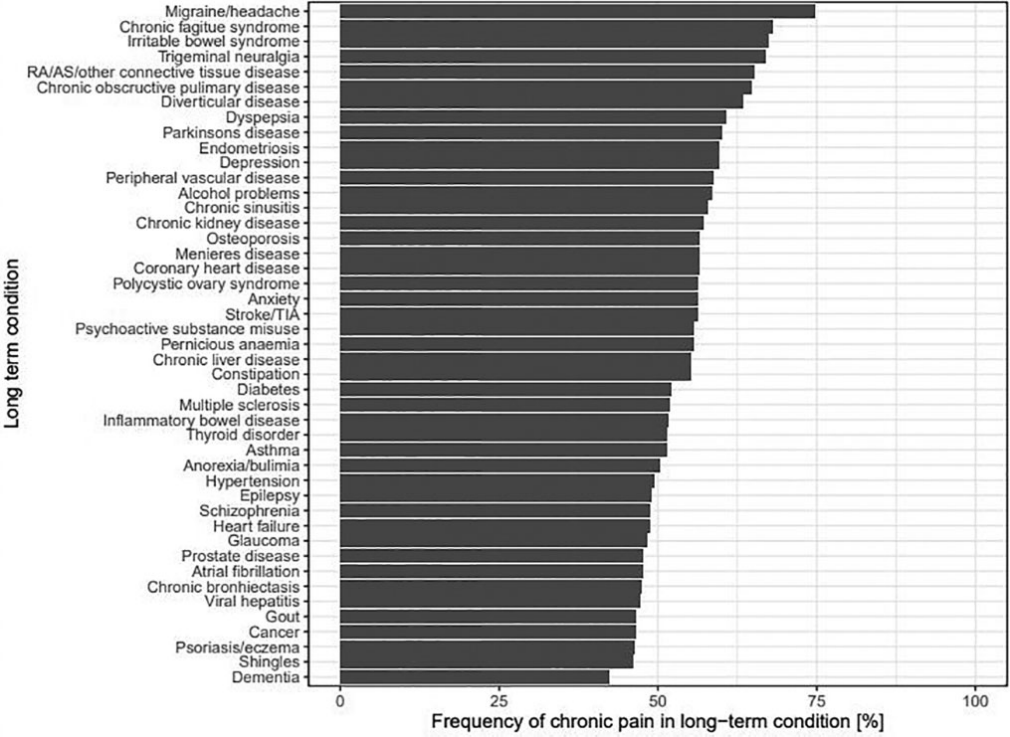
Prevalence of chronic pain in participants with each of the included 45 long-term conditions. RA: rheumatoid arthritis; AS: ankylosing spondylitis.

150,481 participants (69.1%) who reported having chronic pain also reported having one or more LTC, and 78,764 (36.2%) had two or more LTCs. Figure 1 shows each LTC examined and the frequency of participants within that LTC category reporting chronic pain (in at least one site). Notably, chronic pain was highly prevalent in participants with LTCs: in 31 of the 45 LTCs measured, half or more of participants reported experiencing chronic pain. In participants with multimorbidity, 55.6% (N = 76,376) also reported chronic pain. Individually, the highest prevalence of chronic pain appeared predominantly in people with LTCs that are recognised as having pain as a predominant symptom. Participants reporting migraine/headaches had the largest frequency of chronic pain (74.8%, of which 91.6% reported chronic head pain), followed by chronic fatigue syndrome (68.1%, of which 91.1% reported chronic back pain), irritable bowel syndrome (67.4%, of which 90.0% reported chronic knee pain). However, chronic pain was very common in other types of LTCs, including diseases of the digestive system (diverticular disease (63.4%; of which 91.8% reported chronic knee pain); dyspepsia (60.7%; of which 92.2% reported chronic knee pain)) and mental health based LTCs (depression (59.6%; of which 90.2% reported chronic knee pain), and alcohol problems (58.6%; of which 95.4% reported chronic hip pain). Interestingly, participants with some individual LTCs that are known to have acute pain symptoms such as gout, reported a lower frequency of chronic pain (46.6%).

**Figure 2.**
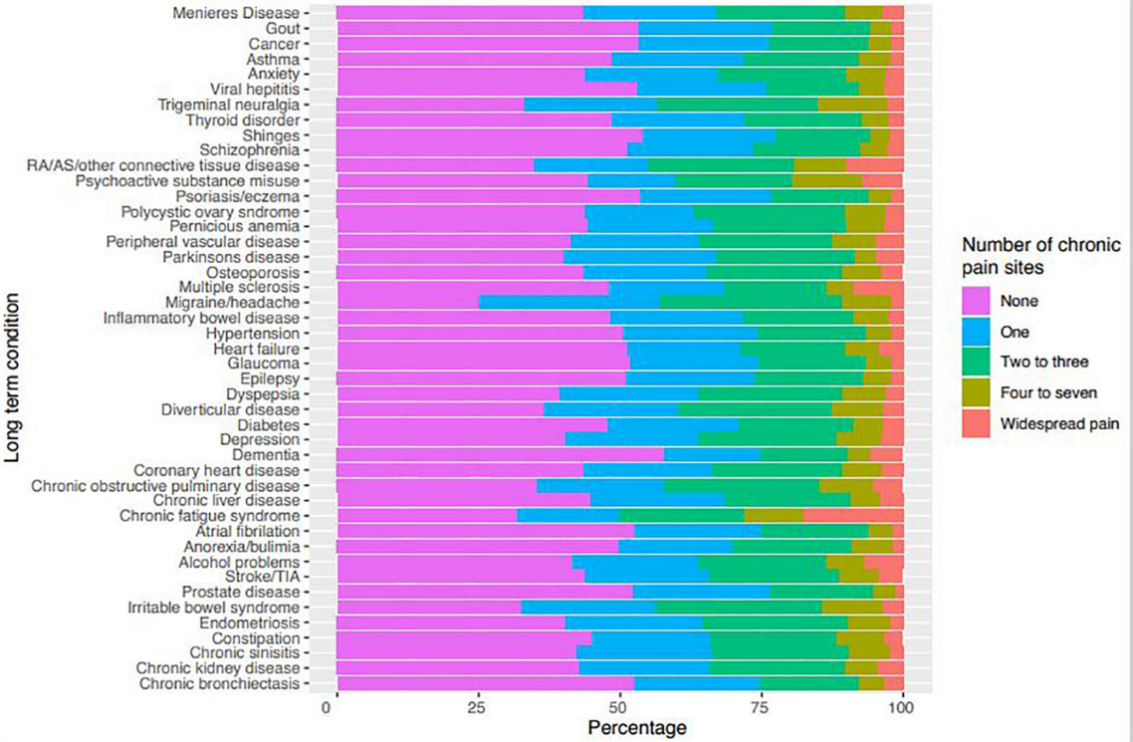
Number of chronic pain sites in each LTC. RA: rheumatoid arthritis; AS: ankylosing spondylitis.

We next measured the relationship between the extent of chronic pain reported and type of LTC by examining the number of sites of chronic pain reported by participants with particular LTCs (Figure 2). Participants with chronic fatigue syndrome had the highest prevalence of widespread pain, with 17.6% reporting this. Widespread pain was also commonly reported by participants with connective tissue diseases (10.0%), multiple sclerosis (8.8%) and psychoactive substance misuse (7.2%). Reporting of four to seven sites of chronic pain were most prevalent in participants with psychoactive substance misuse (12.4%) and chronic fatigue syndrome (10.4%).

### LTCs and risk of chronic pain

To investigate the relationship between the presence of chronic pain and the extent of multimorbidity experienced, we examined the relative risk ratio (RRR) of chronic pain status across the LTC count categories using multinomial logistic regression (Table 2). These models controlled for age, sex, Townsend score, smoking status, alcohol intake, BMI, and physical activity level. There was a dose-based relationship observed between the presence of chronic pain and LTC count: a 36% increased risk of chronic pain was observed in those with a single LTC (RRR 1.36, confidence intervals (CI) 1.34–1.38), an 88% increased risk for those with two to three LTCs (RRR 1.88, CI 1.85–1.91) and more than three times the risk for those with four or more LTCs (RRR 3.56, CI 3.44–3.68) compared to those with no LTCs.

**Table 2.** Multinomial logistic regression model showing relative risk of chronic pain sites in participants with 0, 1, 2–3 and ≥4 long-term conditions. All p-values were two sided and <0.01.

Outcome variable	Presence of chronic pain (RRR (95% CI) Model 1: adjusted for age, sex, Townsend score (deprivation)	Presence of chronic pain (RRR (95% CI) Model 2: Model 1+ smoking, alcohol, BMI and physical activity levels
Number of long-term conditions	Have chronic pain	
0	1	1
1	1.43 (1.43–1.45)	1.36 (1.34–1.38)
2–3	2.13 (2.10–2.16)	1.88 (1.85–1.91)
≥4	4.56 (4.41–4.71)	3.56 (3.44–3.68)

To further elucidate the relationship between LTC count and chronic pain, we carried out a multinomial logistic regression of LTC count and number of pain sites (extent of chronic pain, shown in Table 3) controlling for demographic and lifestyle variables as described above. As with chronic pain presence, there was a dose-based relationship between multimorbidity count and number of sites of chronic pain. This was most apparent in the widespread pain group, where having a LTC count ≥4 increased the risk of widespread pain by over 20 times (RRR 20.13, CI 18.26–22.19) compared with participants with no LTCs.

**Table 3.** Multinomial logistic regression model showing relative risk of number of chronic pain sites in participants with 0, 1, 2–3 and ≥4 long-term conditions. Model 1: adjusted for age, sex, Townsend score (deprivation). Model 2: Model 1 + smoking, alcohol, BMI and physical activity levels. All p-values were two sided and <0.01.

Outcome variable	Extent of chronic pain (RRR (95% CI)							
Number of long-term conditions	1 site		2–3 sites		4–7 sites		Widespread pain	
	Model 1	Model 2	Model 1	Model 2	Model 1	Model 2	Model 1	Model 2
0	1	1	1	1	1	1	1	1
1	1.27 (1.25–1.29)	1.23 (1.21–1.25)	1.59 (1.56–1.62)	1.50 (1.47–1.53)	2.28 (2.16–2.40)	2.07 (1.96–2.19)	2.84 (2.61–3.08)	2.46 (2.26–2.68)
2–3	1.55 (1.52–1.58)	1.44 (1.41–1.47)	2.58 (2.52–2.69)	2.29 (2.24–2.34)	6.04 (5.74–6.34)	4.73 (4.50–4.99)	7.66 (7.08–8.27)	5.50 (5.07–5.97)
≥4	2.18 (2.09–2.28)	1.91 (1.83–2.00)	5.54 (5.33–6.01)	4.45 (4.27–4.64)	25.06 (23.49–26.74)	15.95 (14.90–17.08)	36.59 (33.39–40.09)	20.13 (18.26–22.19)

## Discussion

Our study has shown that chronic pain is extremely common across a wide range of LTCs (N = 45), with chronic pain being reported in more than 50% of people with 31 different LTCs. Over half (53.4%) of participants with 2–3 LTCs and almost three quarters of those with ≥4 LTCs (71.5%) reported at least one site of chronic pain, which was twice and four times more likely than people with no LTCs, respectively. When examining individual types of LTCs co-occurring with chronic pain, we found that it was highly prevalent in both physical LTCs, e.g. migraine, where over 70% of participants reported chronic pain, and mental health based LTCs such as depression and anxiety, which showed over half of participants reporting chronic pain. The relationship between chronic pain status, the number of chronic pain sites and LTC count persisted when controlling for the lifestyle and demographic factors of participants. When examining the relationship with the extent of chronic pain, there was a strong relationship between LTC count and chronic pain; participants with ≥4 LTCs were around 16 times more likely to have chronic pain in between four and seven sites, and 20 times as likely to have chronic widespread pain throughout the body. Thus, there is strong evidence for the relationship between both the presence and extent of chronic pain and the number of LTCs experienced.

The results presented in this study expand on current literature on the relationship between LTCs and chronic pain. Previous research has shown that there is an association between both physical and mental health based individual LTCs and chronic pain. Our work concurs with existing literature that shows that chronic pain is common in people with LTCs that report pain as the primary symptom (such as migraine, chronic fatigue and irritable bowel syndrome), and mental health conditions. However, we also show here that it is also commonly associated with several digestive system related LTCs such as dyspepsia. Previous studies have examined migraines and specific sites of chronic pain, highlighting its co-occurrence with specific sites of chronic pain, including low back pain.^22^ When examining mental health LTCs, we found one UK Biobank study that examined chronic pain, major depression and bipolar disorder, it reported a strong relationship between these conditions and both presence and extent of chronic pain.^14^ A nationally representative sample of patients experiencing mood and anxiety disorders found a significant relationship between the presence of chronic pain and mood and anxiety disorders, particularly panic disorder and post-traumatic stress disorder.^23^ Further, one study by Nicholl et al. described the positive relationship between onset of chronic widespread pain and psychosocial factors such as sleep problems, anxiety and depression.^24^ Previous research has highlighted the impact of chronic pain in conditions where fatigue is a major symptom, particularly in patients diagnosed with fibromyalgia^25,26^ or chronic fatigue syndrome.^27,28^

To date, no general population analyses exist showing the relationship between the number of sites of chronic pain and the degree of multimorbidity or the range of individual LTCs presented here. We were able to find only a single study that characterised the relationship between chronic pain and multimorbidity. Scherer et al.^18^ presented results from a study of 3189 chronic pain patients in primary care aged 65 or older, showing that the level of chronic pain was positively associated with presence of chronic gastritis, hyperuricemia/gout, cardiac insufficiency, neuropathies and depression. However, this paper only examined 8 LTCs, and represents results in an elderly population only. We for the first time here highlight a strong association between increasing number of LTCs and both the presence and extent of chronic pain using a large number of LTCs and in a population aged between 37 and 73 years old. Our results show that chronic pain is an important factor for consideration in the clinical management of patients with specific LTCs and/or multimorbidity.

A clear strength of this study is the large size of the cohort, UK Biobank provides data on over half a million people with reports of a broad range of LTCs (N = 45) and chronic pain. In addition, details on a comprehensive list of potential confounding variables (age, sex, socioeconomic deprivation (as measured using the Townsend score), smoking status, frequency of alcohol intake, BMI and physical activity) were present and adjusted for in our models.

There are some limitations to this study. LTCs were based on self-report data as given by participants and may be under or over-reported and we do not know about the severity of each LTC. Further, we were restricted on how chronic pain was assessed in UK Biobank; in particular on the specific sites of pain, and we did not have information about pain intensity or interference, which is a limitation. While we had data on seven sites of pain as well as widespread pain the use of a regional pain scale^29^ would have allowed a more detailed examination of pain locations. Further, our classification of ‘pain all over the body’ as *widespread* pain, making it distinct from those with 4–7 sites of chronic pain, was based on an understanding that participants who selected this option feel differently about their pain than those who selected separate sites of pain; this classification has been used previously.^14,30^ Chronic pain is a condition in its own right^31^ and can be independent of other LTCs experienced; however, it may be that particular LTCs are associated with the development of chronic pain or vice versa, as is well researched for mood problems, such as depression.^32,33^ In this cross-sectional study we have no information on the temporal nature of chronic pain and LTC development to investigate this issue further. Finally, UK Biobank is a selected population, and is not representative of the wider UK general population. Participants in UK Biobank are known to be mostly White British and comparatively less socioeconomically deprived than the UK average. This may mean that our estimates of the prevalence of chronic pain in those with specific LTCs or multimorbidity is likely to be conservative. However, this work is important and is the first to highlight the prevalence of pain in people living with multimorbidity.

The recently published NICE guidelines on multimorbidity highlight that healthcare providers should be alert to the possibility of chronic pain in patients, and stresses a need to examine whether the patients existing pain management is appropriate.^34^ The impact of chronic pain on an individual’s quality of life^35^ and on society^36^ is well established yet the impact of living with an additional LTC or multimorbidity alongside chronic pain is less well understood and explored. Our study suggests that chronic pain is a key factor for consideration in the clinical management of patients with LTCs and multimorbidity. Further research is required to explore the impacts, if any, on health outcomes including the effect of chronic pain and widespread pain on hospitalisations and mortality in people with multimorbidity.

## Conclusions

This study represents the first examination of the prevalence of chronic pain in participants with a broad range of LTCs and differing levels of multimorbidity. We have highlighted a much-neglected area, namely the co-existence of chronic pain with multimorbidity and specific LTCs, that demands both research and clinical consideration. It is vital to understand the impact of chronic pain on health-related outcomes in order to inform future management of patients who experience chronic pain alongside single or multiple LTCs.

## Supplemental material

Supplemental Material, sj-docx-1-cob-10.1177_26335565211005870 - Prevalence of chronic pain in LTCs and multimorbidity: A cross-sectional study using UK Biobank Click here for additional data file. Supplemental Material, sj-docx-1-cob-10.1177_26335565211005870 for Prevalence of chronic pain in LTCs and multimorbidity: A cross-sectional study using UK Biobank by Ross McQueenie, Bhautesh Dinesh Jani, Stefan Siebert, Philip McLoone, Colin McCowan, Sara Macdonald, Frances S Mair and Barbara I Nicholl in Journal of Comorbidity
