# Supplementary material for: Prevalence of chronic pain in LTCs and multimorbidity: A cross-sectional study using UK Biobank
Source: J Multimorb Comorb. 2021 Dec 21;11:26335565211005870. doi: 10.1177/26335565211005870 (PMC8728767; doi:10.1177/26335565211005870)
Supplement: Supplemental Material, sj-docx-1-cob-10.1177_26335565211005870 - Prevalence of chronic pain in LTCs and multimorbidity: A cross-sectional study using UK Biobank [file sj-docx-1-cob-10.1177_26335565211005870.docx]

| **Name of long-term condition** |
| --- |
| Migraine/headache |
| Chronic fatigue syndrome |
| Irritable bowel syndrome |
| Trigeminal neuralgia |
| Rheumatoid arthritis/ankylosing spondylitis/other connective tissue disorder |
| Chronic obstructive pulmonary disease |
| Diverticular disease |
| Dyspepsia |
| Parkinson’s disease |
| Depression |
| Endometriosis |
| Peripheral vascular disease |
| Alcohol problems |
| Chronic sinusitis |
| Chronic kidney disease |
| Meniere’s disease |
| Osteoporosis |
| Coronary heart disease |
| Polycystic ovary syndrome |
| Anxiety |
| Stroke/transient ischemic attack |
| Psychoactive substance misuse |
| Pernicious anaemia |
| Chronic liver disease |
| Constipation |
| Diabetes |
| Multiple sclerosis |
| Inflammatory bowel disease |
| Thyroid disorder |
| Asthma |
| Anorexia/bulimia |
| Hypertension |
| Epilepsy |
| Schizophrenia |
| Heart failure |
| Glaucoma |
| Prostate disease |
| Atrial fibrillation |
| Chronic bronchiectasis |
| Viral hepatitis |
| Gout |
| Cancer |
| Psoriasis/Eczema |
| Shingles |
| Dementia |

**Supplementary Table 1 – List of conditions included in long-term condition count.**
